# Parental Exposure to Pesticides and Childhood Brain Cancer: U.S. Atlantic Coast Childhood Brain Cancer Study

**DOI:** 10.1289/ehp.0800209

**Published:** 2009-02-13

**Authors:** Youn K. Shim, Steven P. Mlynarek, Edwin van Wijngaarden

**Affiliations:** 1 Division of Health Studies, Agency for Toxic Substances and Disease Registry, Atlanta, Georgia, USA; 2 Department of Occupational and Environmental Health, College of Public Health, University of South Florida, Tampa, Florida, USA; 3 Department of Community and Preventive Medicine, University of Rochester Medical Center, Rochester, New York, USA

**Keywords:** astrocytoma, brain cancer, children, parental exposure, pesticides, PNET

## Abstract

**Background:**

The etiology of childhood brain cancer remains largely unknown. However, previous studies have yielded suggestive associations with parental pesticide use.

**Objectives:**

We aimed to evaluate parental exposure to pesticides at home and on the job in relation to the occurrence of brain cancer in children.

**Methods:**

We included 526 one-to-one–matched case–control pairs. Brain cancer cases were diagnosed at < 10 years of age, and were identified from statewide cancer registries of four U.S. Atlantic Coast states. We selected controls by random digit dialing. We conducted computer-assisted telephone interviews with mothers. Using information on residential pesticide use and jobs held by fathers during the 2-year period before the child’s birth, we assessed potential exposure to insecticides, herbicides, and fungicides. For each job, two raters independently classified the probability and intensity of exposure; 421 pairs were available for final analysis. We calculated odds ratios (ORs) and 95% confidence intervals (CIs) using conditional logistic regression, after adjustment for maternal education.

**Results:**

A significant risk of astrocytoma was associated with exposures to herbicides from residential use (OR = 1.9; 95% CI, 1.2–3.0). Combining parental exposures to herbicides from both residential and occupational sources, the elevated risk remained significant (OR = 1.8; 95% CI, 1.1–3.1). We observed little association with primitive neuroectodermal tumors (PNET) for any of the pesticide classes or exposure sources considered.

**Conclusions:**

Our observation is consistent with a previous literature reporting suggestive associations between parental exposure to pesticides and risk of astrocytoma in offspring but not PNET. However, these findings should be viewed in light of limitations in exposure assessment and effective sample size.

Brain cancers are the second most common cancer in children, but little is known about their etiology. Some genetic conditions (Barker et al. 1987; Gorlin et al. 1965; Hamilton et al. 1995; Li et al. 1988; Rouleau et al. 1987) and ionizing radiation (Karlsson et al. 1997; Ron et al. 1988) are the only accepted causes of childhood brain cancer. Parental occupation and residential activities are sources of exposure to carcinogens, neurotoxins, and microorganisms that could also increase risk. Although parental occupational exposures have been a focus of some research on childhood brain cancer, the studies have generally been limited by small sample sizes, heterogeneous case groups, and crude exposure assessment. Furthermore, these studies have rarely considered exposures from multiple sources. Despite these methodological limitations, epidemiologic studies have yielded suggestive associations with farm residence and pesticide use (Olshan and van Wijngaarden 2003).

In this population-based case–control study we evaluated the association between the occurrence of brain cancer in children and parental exposure to pesticides in occupational and residential settings.

## Methods

### Study population

Selection of cases and controls has been described previously (Agency for Toxic Substances and Disease Registry 2004; Choi et al. 2006), and is summarized here. Briefly, cases eligible for the original Atlantic Coast childhood brain cancer study included all incident cases of first primary brain cancer (*International Classification of Diseases for Oncology*, topography codes C71.0–C71.9, including all morphologic codes with a behavior code of 3, excluding lymphomas) (Percy et al. 1990). Cases were diagnosed at < 10 years of age between 1993 and 1997, were born in the United States, and were residents of one of the four states (Florida, New Jersey, New York excluding New York City, and Pennsylvania) at the time of diagnosis. New York City was excluded mainly because the unique characteristics of the city make tracing cases and identifying controls very difficult. In addition, an eligible case had to have a telephone in the household and have the biological mother available for an interview in English. Of the 937 case children initially identified by the four statewide cancer registries, we screened 709 for eligibility and found 662 to be eligible. We completed interviews for 526 cases (56.1% of the originally identified 937 cases or 79.5% of the 662 eligible case children) after excluding nine children for whom matched controls could not be identified.

We selected controls by random digit dialing (RDD) (Wacholder et al. 1992; Waksberg 1978) and individually matched them to cases by sex, race (white, black, or other), birth year (± 1 year), and state of residence at the time of case diagnosis. However, as control selection came close to the end, sex was dropped from the matching criteria for 10 cases to ease the effort to find a matched control. This allowed us to use as much information as we could, as previously recommended (Greenland 1986). We used age at diagnosis for each case as a reference age for the corresponding control. Eligible controls had to be free of cancer, be born in the United States, and have the biological mother available for a telephone interview in English. In a household with more than one eligible child, we selected the younger child as a control. Among the 20,802 working residential numbers identified from the RDD sample, each of 3,553 households had a child meeting the eligibility criteria. Of the 3,553 children, 820 met the matching criteria. One hundred twenty-two eligible controls did not have a matching case available, and 526 agreed to participate. The overall response rate was 62%, with screening rate of 88% and participation rate of 70%.

Mothers gave their consent to participate in the study over the telephone. For both case and control mothers, bilingual (Spanish) interviewers were available to assist mothers who spoke English as their second language. We excluded only mothers who spoke no English and could not communicate in English with the assistance. The study protocol was approved by the Centers for Disease Control and Prevention and four state institutional review boards.

### Data collection

We interviewed the biological mothers of cases and controls via a computer-assisted telephone interview system over 13 months between 2000 and 2001. We conducted the interviews sequentially by state to reduce the differences in the exposure–interview lag time between the case and control in each pair. The average time to complete the interview was 54 min.

We developed the study questionnaire by modifying the questionnaire used in previous studies of childhood cancer (Agency for Toxic Substances and Disease Registry 2004). Briefly, the questionnaire sought information about demographics and exposure, including parents’ ages at child’s birth, mother’s education level, household income, residential history, residential use of pesticides, and parents’ occupational histories.

To assess the residential exposure to pesticides, we asked mothers whether their household used insecticides, herbicides, or fungicides in their gardens and lawns during the 2 years before the child’s birth. If so, we also asked who in the household treated the lawns and gardens.

We took parents’ job histories covering the 2 years before the child’s birth from each mother. For each job that mothers held, they were asked about the type of company or industry, occupation or job title, main job or activity, year starting and ending the job, and full-time or part-time status. Other information asked about each job included whether they were exposed to specific groups of chemicals, including pesticides. We also asked each mother to provide the biological father’s job information, including the type of company or industry, occupation or job title, main job or activity, year starting and ending the job, and full-time or part-time status.

### Exposure assessment

We coded occupation and industry using the 1990 U.S. Bureau of the Census classification system of jobs and industries (U.S. Bureau of the Census 1992). We relied primarily on occupation and industry and, to a lesser extent, ancillary job information to assign exposure levels. We evaluated four broad classes of pesticides: insecticides, herbicides, and agricultural and nonagricultural fungicides. “Nonagricultural fungicides” is the term used for disinfectants, germicides, and other chemicals used to control bacteria and other organisms. Evaluation of specific chemicals or chemical structures was not feasible. Two raters independently reviewed the jobs. One rater (E.V.W.) was trained by an industrial hygienist to conduct an exposure assessment specifically for a previous study of occupational pesticide exposure and childhood brain cancer (van Wijngaarden et al. 2003), whereas the other rater (S.P.M.) is a certified industrial hygienist who specializes in exposure assessment, including pesticide exposures. For each job, these raters classified the probability and intensity of exposure based on an extensive industrial hygiene literature review of determinants and levels of pesticide exposure and a job–exposure matrix created previously (Ji et al. 2001; van Wijngaarden et al. 2003). We assigned probability, defined as the percentage of workers in a particular industry exposed to insecticides, herbicides, agricultural fungicides, or nonagricultural fungicides, to one of four categories: < 10%, 10–49%, 50–89%, and ≥90%. We also assigned exposure intensity to one of four levels on a scale from 0 to 3 based on considerations published previously (van Wijngaarden et al. 2003). Because the major route of exposure to pesticides is deposition, we restricted assessment of intensity to dermal exposure (van Hemmen 1992). Finally, we assigned an overall confidence level for each job on a scale of 1 (lowest) to 4 (highest). Raters were blinded to case status.

We consolidated exposure assignments across raters using the following algorithm. First, we calculated rater-specific scores for each job by multiplying intensity and probability. We then grouped these scores into three categories: “none” (product of intensity and probability = 0), “some” (product of intensity and probability = 1 or 2), and “substantial” (product of intensity and probability ≥3). We selected these cutoffs to ensure that each of these groups would have a sufficient number of jobs. For individuals who held multiple jobs, we used an average score to assign the exposure categories. Finally, we compared final grouping between raters. We took no further action if each rater assigned a job to the same category. Jobs that raters assigned to differing groups were categorized using the probability and intensity scores associated with the higher confidence level; when the confidence level was the same, we used an average of the two raters’ probability and intensity scores.

We assessed a total of 1,231 jobs for exposure for fathers and 1,006 jobs for mothers. The agreement between raters in assigning exposure into three categories (i.e., none, some, and substantial) was moderate for fathers according to the previously suggested categorization of kappa (κ) coefficients (Sim and Wright 2005), with a weighted κ of 0.5 for insecticides, 0.5 for herbicides, and 0.6 for agricultural fungicides. For nonagricultural fungicides, the agreement was fair, with a weighted κ of 0.3. For mothers, however, the agreement was mostly low (a weighted κ of 0.3 for insecticides, 0.1 for herbicides, 0.02 for agricultural fungicides, and 0.2 for nonagricultural fungicides).

We assessed residential use of pesticides for gardens and lawns for three broad classes: insecticides, herbicides, and fungicides. We categorized the frequency of use into “ever” and “never.” Information on the use of personal protective equipment was not sufficiently detailed to permit consideration for the occupational exposure assessment.

### Statistical analysis

We performed analyses separately for primitive neuroectodermal tumors (PNET) and astrocytoma, the two most frequent types of childhood brain cancer, to evaluate etiologic heterogeneity. We combined the remaining brain cancer types into the “all other types” category. We carried out conditional logistic regression analyses to estimate odds ratios (ORs) and their 95% confidence intervals (CIs). Of the limited potential confounders examined (mother’s age at child’s birth and mother’s education), mother’s education (≤ high school and > high school) changed the ORs of some exposure variables by ≥ 10%. Therefore, we included mother’s education in all models to capture potential confounding effects broadly related to socioeconomic status. We used SAS software (version 9.1; SAS Institute Inc., Cary, NC) for the analyses.

For residential use of pesticides, we calculated the risk estimates for each of the three types of pesticides, regardless of who applied. We conducted additional analyses separately for mother’s and father’s application of residential pesticides; these analyses were not mutually exclusive because in some households both parents applied pesticides.

After excluding those with missing information on jobs or the covariate (i.e., mother’s education), a total of 421 case–control pairs were available for fathers and 269 pairs for mothers for the data analysis. Because of the low agreement in exposure assessment and small number of case–control pairs available for mothers, in this report we focused primarily on father’s occupational exposures. We also computed risk estimates for pesticides by combining father’s occupational and residential exposures.

## Results

Most children in this study were white, male (except for children with brain tumors other than astrocytoma and PNET), and born between 1988 and 1992 (Table 1). Case children were about equally distributed among the geographic regions included in our study. By definition, the control children had a similar profile. Mothers of case children with astrocytoma and PNET were generally younger and had higher education than mothers of control children (Table 2). Interestingly, mothers of case children with brain tumors other than astrocytoma and PNET seemed to be somewhat older and less educated (Table 2).

We observed patterns of increased risk in relation to residential pesticide use, most consistently for astrocytoma (Table 3). Herbicide use showed an elevated risk of astrocytoma of about 2-fold regardless of which parent used this class of pesticides. We found ORs of similar magnitude for fungicide use, but estimates were considerably less precise and not statistically significant. We examined the effect of precautions taken for residential application of pesticides, by using unconditional logistic regression after controlling for matching variables. Adjusted ORs were significantly lower for fathers who always or usually washed immediately afterward (OR = 0.4; 95% CI, 0.1–1.0) or wore protective clothing (OR = 0.4; 95% CI, 0.2–0.6) during the application, compared with fathers who never or sometimes took these precautions.

Parental exposure to pesticides on the job was considerably less common than for residential use; among all fathers (842 fathers or 421 case–control pairs), any job-related exposure to pesticides was 13.0% for insecticides, 8.4% for herbicides, 5.7% for agricultural fungicides, and 20.3% for nonagricultural fungicides. Consequently, the resulting risk estimates were statistically imprecise for the association with specific childhood brain tumor subtypes. For example, adjusted ORs were 3.0 (95% CI, 0.6–15.0) for astrocytoma and 1.2 (95% CI, 0.2–5.9) for PNET for children whose fathers had potentially substantial exposure to herbicides on the job. Nevertheless, consistent with our findings for residential pesticide use, the results show some indications of an increased risk of astrocytoma and other tumor types, but not PNET, in relation to father’s occupational exposure, as determined by our algorithm. We found no indication of increased risk of brain cancer in children associated with maternal occupational exposure to any of the four types of pesticide classes (data not shown), but results were even more imprecise and the assessment of occupational exposure among mothers was more difficult.

Finally, we evaluated the risk associated with combined use of residential (by anyone, or fathers only) and paternal occupational exposure to pesticides; findings were similar to those reported for residential use only (Table 4).

## Discussion

Many pesticides are carcinogenic to animals, and some are considered carcinogenic to humans with varied degree of evidence. For example, the U.S. Environmental Protection Agency has classified chlordane, heptachlor, tetrachlorvinphos, carbaryl, and propoxur as probable or likely human carcinogens, and lindane, dichlorvos, phosmet, and permethrin as suggestive or possible carcinogens (U.S. Environmental Protection Agency 2003). Maternal and cord blood levels of some pesticides are similar, demonstrating that they are readily transferred from mother to fetus during pregnancy (Whyatt et al. 2003). Parental exposures may act before the child’s conception, during gestation, or after birth to increase the risk of cancer. Before conception, exposures may cause mutations or epigenetic alterations in gene expression, such as genomic imprinting or DNA methylation, in the sperm or egg (Anderson et al. 2000). Exposure after conception (i.e., during the pregnancy or after birth) may cause somatic cell mutations or alterations in hormonal or immunologic function (Daniels et al. 1997) that affect cancer risk (Anderson et al. 2000).

However, potential effects of pesticides on risk for childhood cancers are not clearly understood. Several epidemiologic studies reported risk of childhood brain cancer associated with residential pesticide use with mixed results. Pesticide exposure during pregnancy was not a risk factor for childhood brain cancer in some studies (Bunin et al. 1994; Kuijten et al. 1990; Leiss and Savitz 1995), whereas others reported at least one type of pesticide associated with an increased risk (Pogoda and Preston-Martin 1997; Preston-Martin et al. 1982; Wilkins and Bunn 1997). In one study, more case mothers reported using pesticides, but case and control mothers were similar with respect to whether their homes were treated by an exterminator at any time during pregnancy (Preston-Martin et al. 1982). In another study, bombs or no-pest strips used for nuisance pests during pregnancy were associated with a significant increase in childhood brain cancer risk, but insecticide or herbicide use was not (Wilkins and Bunn 1997).

The epidemiologic literature assessing occupational pesticide exposure in relation to childhood brain cancer was recently reviewed with a focus on fathers (Olshan and van Wijngaarden 2003). Paternal occupational exposure to pesticides as a risk factor for childhood nervous system tumors has been examined in several studies (Cordier et al. 1997; Fear et al. 1998; Feychting et al. 2001; Heacock et al. 2000; Kristensen et al. 1996; Kuijten et al. 1992; McKean-Cowdin et al. 1998; van Wijngaarden et al. 2003; Wilkins and Koutras 1988; Wilkins and Sinks 1990). The evidence for an association between occupational pesticide exposure and childhood brain cancer remains inconclusive after consideration of the literature, but the exposure assessment has generally been crude. Studies that used industrial hygiene expert assessment to estimate the probability of pesticide exposure reported several elevated risks (Feychting et al. 2001; van Wijngaarden et al. 2003), and another study with detailed census data reported an exposure–response relationship with pesticide purchase that was strongest for the tumor category that included PNET (Kristensen et al. 1996). These studies may be considered of better quality than previous studies that relied mostly on job title or industry, and they provide suggestive leads for further research on paternal pesticide exposure and brain cancer in their children.

Maternal occupation has been studied less often with generally less well-defined definitions of exposure (Cordier et al. 1997, 2001; Feychting et al. 2000; Holly et al. 1998; Kuijten et al. 1992; McCredie et al. 1994a, 1994b; McKean-Cowdin et al. 1998; Peters et al. 1981; Sorahan et al. 1999). McCredie et al. (1994a, 1994b) did not find an increased risk related to mother’s farm residence or farm employment, but the recent international case–control study showed a 2-fold increased risk of brain cancer in children of mothers exposed to agricultural pesticides on the job (Efird et al. 2003). Van Wijngaarden et al. (2003) observed an increased risk with insecticide exposure for astrocytomas (OR = 1.9; 95% CI, 1.1–3.3) but no association between PNET and maternal exposure to any pesticide class. The association of childhood brain cancer with farm residence, employment in agriculture, and contact with farm animals could be explained by exposure to pesticides used on crops and animals or exposure to viruses or other microorganisms.

The results of this study must be viewed in light of several possible limitations. First, selection bias can make the interpretation of case–control studies difficult. This is especially true in hospital-based case–control studies, in which it is difficult to identify the source population from which the cases were derived. In our population-based study, this issue appears less of concern because cases were identified by statewide cancer registries and controls were selected from the general population by matching the state of residence accordingly. Nevertheless, control selection on the basis of RDD can lead to selection bias because of potential incomplete phone coverage, residences with multiple phone lines, and nonresponse (Wacholder et al. 1992). It is also possible that this study may have excluded families with high exposures to pesticides, such as migrant farm workers, because of their language barriers. If there truly was an association between pesticides and childhood brain cancer, this would have excluded cases in greater proportion than controls, resulting in selection bias. However, in our study the proportion of potential study participants who were excluded because of language barrier (any language) was relatively small: about 1% of the located cases and about 2% of the potential controls identified through working residential telephone numbers.

Furthermore, recall bias is always of concern in population-based case–control studies of childhood cancer in which parents of case children are more likely to accurately report (or overreport) specific exposures potentially associated with disease compared with parents of healthy children. Moreover, they may report more detailed job histories than do parents of control children (van Wijngaarden et al. 2003). Nevertheless, we primarily relied on standard job and industry titles in our exposure assessment, and we believe differential reporting is unlikely. Additionally, misclassification of exposure certainly occurred, which most likely yielded a conservative bias in the ORs. However, in our analysis we excluded 208 fathers (104 pairs) because either their job information was missing or their matched case or control counterparts had missing job information. This exclusion might have introduced bias, the direction of which is difficult to predict.

Interpretation of our findings of significant risk associated with the father’s herbicide application for lawns and gardens requires caution. In examining the risk by who applied the pesticides, we could not analyze the data after excluding those households that reported the applications by both professionals and parents because the numbers were small. Pesticides applied by professionals may have been more toxic (e.g., pesticides requiring “restricted use”) than those used by parents. Further, we relied on maternal report of residential pesticide use, and the mother’s recall on the father’s application could have been inaccurate. However, although data were limited, we found that ORs were higher among fathers who never or occasionally washed immediately afterward or wore protective clothing, compared with those who always or usually took such precautions.

Two raters evaluated all jobs mothers and fathers held during the 2 years before birth, and the interrater agreement was fair to moderate (κ = 0.3–0.6) for fathers and poor to fair (κ = 0.02–0.3) for mothers, according to a previously proposed categorization of κ coefficients (Sim and Wright 2005). The interrater agreement for fathers is similar to estimates reported elsewhere (van Wijngaarden et al. 2003), although in the present study paternal work history was provided by mothers and subject to possible inaccuracies. The low inter-rater agreement for mother’s job exposures may reflect the lack of research focused on occupational pesticide exposure among jobs more commonly held by women. The main source of discrepancy between raters was the large number of women who were considered minimally exposed by one rater and unexposed by the other rater, especially for clerical and retail jobs. Given the limited work history data available for mothers in this data set, it is unclear which assignment is more accurate, although a recent report of cases of pesticide poisoning in the retail industry suggests that pesticide exposure in these environments may be likely (Calvert et al. 2007).

In conclusion, these data provide some evidence for an association between brain cancer risk in children and paternal exposure to pesticides during the 2 years before birth, in particular for astrocytoma and herbicide exposure. Our findings are consistent with those reported by van Wijngaarden et al. (2003) and Kuijten et al. (1992), although they appear to contradict results published by Bunin et al. (1994) and Kristensen et al. (1996), which showed stronger associations of pesticides with PNET rather than with astrocytoma. Although several suggestions regarding potential biological mechanisms have been made (Olshan and van Wijngaarden 2003), it is currently unclear whether they are more relevant to astrocytoma or PNET.

Future epidemiologic studies investigating environmental risk factors of childhood brain cancer could benefit from close collaboration with other scientific disciplines. Developing biomarkers—both of exposure and of early health effects—that can be measured reliably should help future studies. In addition, future studies should consider examining potential gene–environment interactions, as the candidate genes involved in the chemical metabolism become known, because the metabolism of environmental chemicals may vary between individuals because of genetic polymorphisms.

## Figures and Tables

**Table 1:** Distribution of case children’s demographic characteristics used as matching variables for control selection, by histopathologic type of childhood brain cancer [*n* (%)].

Characteristic^a^	Astrocytoma cases	PNET cases	All other cases
Total	226 (100)	146 (100)	154 (100)

Race			

White	198 (87.6)	131 (89.7)	126 (81.8)
African American	26 (11.5)	12 (8.2)	22 (14.3)
Other	2 (0.9)	3 (2.1)	6 (3.9)

Birth year			

1983–1987	52 (23.0)	30 (20.6)	29 (18.8)
1988–1992	138 (61.1)	78 (53.4)	86 (55.8)
1993–1997	36 (15.9)	38 (26.0)	39 (25.3)

Sex			

Male	141 (62.4)	98 (67.1)	73 (47.4)
Female	85 (37.6)	48 (32.9)	81 (52.6)

Resident state at reference age			

Florida	65 (28.8)	46 (31.5)	40 (26.0)
New Jersey	45 (19.9)	30 (20.6)	41 (26.6)
New York	55 (24.3)	32 (21.9)	27 (17.5)
Pennsylvania	61 (27.0)	38 (26.0)	46 (29.9)

a One control was selected for each case by matching on sex, birth year ± 1 year, race, and resident state at reference age (i.e., age at case diagnosis). Sex was dropped from the matching criteria for 10 cases to ease the effort to find a matched control.

**Table 2:** Demographic and socioeconomic characteristics of the parents, by histopathologic type of childhood brain cancer [*n* (%)].

	Astrocytoma		PNET		All other types	
Characteristic	Cases	Controls	Cases	Controls	Cases	Controls
Total	226 (100)	226 (100)	146 (100)	146 (100)	154 (100)	154 (100)

Father’s age at child’s birth (years)						

< 20	6 (2.7)	6 (2.7)	7 (4.8)	1 (0.7)	4 (2.6)	4 (2.6)
20–34	165 (73.0)	147 (65.0)	96 (65.8)	99 (67.8)	103 (66.9)	98 (63.6)
≥ 35	46 (20.4)	55 (24.3)	33 (22.6)	38 (26.0)	43 (27.9)	39 (25.3)
Unknown	9 (4.0)	18 (8.0)	10 (6.9)	8 (5.5)	4 (2.6)	13 (8.4)

Mother’s age at child’s birth (years)						

< 20	13 (5.8)	8 (3.5)	10 (6.9)	6 (4.1)	9 (5.8)	15 (9.7)
20–34	185 (81.9)	176 (77.9)	111 (76.0)	110 (75.3)	110 (71.4)	106 (68.8)
≥ 35	28 (12.4)	38 (16.8)	25 (17.1)	29 (19.9)	35 (22.7)	31 (20.1)
Unknown	0 (0)	4 (1.8)	0 (0)	1 (0.7)	0 (0)	2 (1.3)

Household income per year						

≤ $2,000	26 (11.5)	35 (15.5)	23 (15.8)	15 (10.3)	29 (18.8)	17 (11.0)
$2,001–$50,000	73 (32.3)	62 (27.4)	50 (34.2)	49 (33.6)	45 (29.2)	46 (29.9)
> $50,000	105 (46.5)	112 (49.6)	63 (43.2)	73 (50.0)	70 (45.5)	75 (48.7)
Unknown	22 (9.7)	17 (7.5)	10 (6.8)	9 (6.2)	10 (6.5)	16 (10.4)

Mother’s education level						

≤ High school	65 (28.8)	85 (37.6)	44 (30.1)	52 (35.6)	60 (39.0)	46 (29.9)
College	134 (59.3)	114 (50.4)	78 (53.4)	78 (53.4)	76 (49.4)	94 (61.0)
Postcollege	26 (11.5)	27 (12.0)	24 (16.4)	16 (11.0)	18 (11.7)	14 (9.1)
Unknown	1 (0.4)	0 (0)	0 (0)	0 (0)	0 (0)	0 (0)

**Table 3:** Parental lawn and garden pesticide use during the 2-year period before the child’s birth and occurrence of childhood brain cancer (no. of discordant pairs).^a^

	Astrocytoma			PNET			All other types		
Pesticides	+/−	−/+	OR^b^ (95% CI)	+/−	−/+	OR^b^ (95% CI)	+/−	−/+	OR^b^ (95% CI)
Ever used pesticides for gardens and lawns^c^									

Insecticides	52	39	1.3 (0.9–2.0)	25	23	1.1 (0.6–1.9)	26	23	1.2 (0.7–2.0)
Herbicides	53	27	1.9 (1.2–3.0)	26	24	1.0 (0.6–1.8)	25	28	1.0 (0.6–1.8)
Fungicides	13	7	1.8 (0.7–4.6)	8	6	1.3 (0.5–3.8)	11	5	2.6 (0.9–7.6)

Father applied pesticides for gardens and lawns									

Insecticides	28	26	1.0 (0.6–1.8)	20	19	1.0 (0.5–1.9)	20	10	2.3 (1.0–5.0)
Herbicides	40	20	2.0 (1.2–3.4)	21	18	1.1 (0.5–2.0)	15	18	1.0 (0.5–2.0)
Fungicides	3	1	3.1 (0.3–30.0)	4	1	3.6 (0.4–32.6)	8	3	3.3 (0.9–13.0)

Mother applied pesticides for gardens and lawns									

Insecticides	18	18	1.0 (0.5–1.9)	6	8	0.7 (0.3–2.2)	10	12	0.9 (0.4–2.2)
Herbicides	13	7	1.9 (0.7–4.8)	6	7	0.8 (0.3–2.5)	5	13	0.4 (0.1–1.1)
Fungicides	10	6	1.7 (0.6–4.8)	5	3	1.6 (0.4–6.9)	9	3	3.4 (0.9–12.6)

a The total numbers of discordant case–control pairs where “case used (+)/control not used (−)” and “case not used (−)/control used (+).”

b ORs and 95% CIs were calculated by conditional logistic regression for each class of pesticide use (ever vs. never), adjusted for mother’s education level (≤ high school vs. > high school).

c Pesticide applications by anyone, including mother, father, professionals, and others.

**Table 4:** Combination of residential use of and paternal occupational exposure to pesticides during the 2-year period before the child’s birth and occurrence of childhood brain cancer (no. of discordant pairs).^a^

	Astrocytoma			PNET			All other types		
Pesticides	+/−	−/+	OR^b^ (95% CI)	+/−	−/+	OR^b^ (95% CI)	+/−	−/+	OR^b^ (95% CI)
Residential use^c^ and/or potential substantial exposure through father’s job									

Insecticides	50	34	1.5 (0.9–2.3)	25	22	1.2 (0.6–2.1)	29	22	1.3 (0.8–2.3)
Herbicides	52	26	1.9 (1.2–3.1)	25	21	1.2 (0.6–2.1)	26	28	1.1 (0.6–1.9)
Fungicides	18	10	1.8 (0.8–4.0)	13	10	1.4 (0.6–3.2)	5	8	2.0 (0.8–4.7)

Father’s application in residence and/or potential substantial exposure through father’s job									

Insecticides	31	27	1.1 (0.7–1.9)	22	19	1.2 (0.6–2.2)	25	10	2.9 (1.4–6.2)
Herbicides	41	22	1.8 (1.1–3.1)	22	18	1.1 (0.6–2.2)	18	20	1.0 (0.5–2.0)
Fungicides	12	6	2.1 (0.8–5.5)	9	6	1.6 (0.6–4.4)	14	7	2.1 (0.8–5.3)

a The total numbers of discordant case–control pairs where “case used (+)/control not used (−)” and “case not used (−)/control used (+).”

b ORs and 95% CIs were calculated by conditional logistic regression for each class of pesticide use (ever vs. never), adjusted for mother’s education level (≤ high school vs. > high school).

c Pesticide applications by anyone, including mother, father, professionals, and others.
